# The Role of Ultrasound Versus Radiography in Knee Osteoarthritis: A Bilateral Comparative Study

**DOI:** 10.3390/life16091453

**Published:** 2026-08-31

**Authors:** Mihaela-Amalia Harosa, Andreea-Iulia Nicoară, Andreea Lili Bărbulescu, Cristina-Elena Biță, Ștefan-Cristian Dinescu, Emilio Filippucci, Francesco Porta, Florentin Ananu Vreju

**Affiliations:** 1 Doctoral School, University of Medicine and Pharmacy of Craiova, 2 Petru Rareș Street, 200349 Craiova, Romania; 2 Radiology and Medical Imaging Laboratory, Craiova Emergency County Hospital, 1 Tabaci Street, 200642 Craiova, Romania; 3 Department of Pharmacology, Faculty of Medicine, University of Medicine and Pharmacy of Craiova, 2 Petru Rareș Street, 200349 Craiova, Romania; 4 Department of Rheumatology, University of Medicine and Pharmacy of Craiova, 2 Petru Rareș Street, 200349 Craiova, Romania; 5 Rheumatology Unit, Polytechnic University of Marche, Via Aldo Moro 25, 60035 Jesi, Ancona, Italy; 6 Interdisciplinary Pain Medicine Unit, Santa Maria Maddalena Hospital, Via Gorizia 2, 45030 Occhiobello, Rovigo, Italy; 7 Rheumatology Research Center, University of Medicine and Pharmacy of Craiova, 2 Petru Rareș Street, 200349 Craiova, Romania

**Keywords:** knee osteoarthritis, musculoskeletal ultrasonography, radiography, meniscal extrusion, joint space narrowing, marginal osteophytes, joint effusion

## Abstract

Knee osteoarthritis (OA) is traditionally evaluated using conventional radiography (Rx), which is limited to assessing structural bone changes and provides only an indirect measure of cartilage status through joint space width (JSW). Musculoskeletal ultrasonography (US) allows direct visualization of cartilage, marginal osteophytes, meniscal extrusion, and joint effusion, but its added value over standard radiography in bilateral knee assessment remains incompletely characterized. This observational, cross-sectional study prospectively included 30 symptomatic patients (60 knees; 24 women; median age 69 years, range 56–85) with clinically diagnosed knee OA, all evaluated by bilateral weight-bearing radiography and standardized US. Non-parametric tests were used (*p* < 0.05). Ultrasound detected marginal osteophytes more frequently than radiography in both knees (86.7% versus 56.7% on the left, *p* = 0.003; 83.3% versus 63.3% on the right, *p* = 0.014), with no osteophyte identified by radiography alone. Anterior femoral US cartilage thickness did not correlate with radiographic tibiofemoral medial JSW. Meniscal extrusion was associated with medial JSW narrowing in the right knee (*p* = 0.024), and joint effusion occurred only in knees with ultrasound-detected osteophytes (*p* = 0.003 both knees). Ultrasonography provides complementary structural and mechano-inflammatory information beyond radiography and may support a multiparametric characterization of knee OA.

## 1. Introduction

Knee osteoarthritis (OA) is one of the most prevalent musculoskeletal disorders worldwide and a major cause of chronic pain, functional limitation and disability in adults. Its increasing burden is closely related to population aging, obesity, and physical inactivity, making knee OA a growing clinical and socioeconomic challenge [1,2]. Although the disease has traditionally been regarded as a degenerative cartilage disorder, current evidence supports a broader concept of OA as a whole-joint disease involving the articular cartilage, subchondral bone, synovium, menisci, ligaments and periarticular soft tissues [3].

Conventional radiography remains the most widely used imaging method for the diagnosis and structural grading of knee OA. Radiographic assessment provides important information regarding joint space narrowing (JSN), marginal osteophytes, subchondral sclerosis and compartmental involvement. However, several important limitations affect its diagnostic performance. Its two-dimensional projection may underestimate early marginal bone remodeling, and radiographic joint space width (JSW) does not exclusively reflect cartilage thickness, as it may also be influenced by meniscal position, meniscal extrusion, lower-limb alignment and other biomechanical factors [4,5]. These limitations have prompted increasing interest in complementary imaging methods.

Musculoskeletal ultrasonography (US) has increasingly gained attention as a non-invasive, accessible, and radiation-free imaging method capable of assessing multiple components of knee OA. Beyond the indirect structural information provided by radiography, ultrasound allows direct visualization of superficial marginal osteophytes, measurement of anterior femoral cartilage thickness, detection of meniscal extrusion and identification of joint effusion or synovial abnormalities [6,7]. These features are particularly relevant because they capture both structural and inflammatory aspects of the disease, supporting a more comprehensive imaging evaluation of knee OA.

Previous comparative studies have suggested that US may detect certain osteoarthritic features, particularly marginal osteophytes, more frequently than conventional radiography [8,9]. In addition, ultrasound assessment of meniscal extrusion has become increasingly relevant because of its strong association with JSN, cartilage loss and structural progression [10]. Okano et al. assessed 84 consecutive patients (166 knees) attending a single rheumatology outpatient clinic and reported strong correlations between US and radiographic osteophyte grades (medial *ρ* = 0.752; lateral *ρ* = 0.695). US identified osteophytes in 20 of 46 knees graded 0 medially and in 26 of 66 knees graded 0 laterally on radiography [8]. Suwal et al. examined 136 patients recruited in a radiology department and found a higher mean osteophyte count on US than on radiography (4.04 ± 1.96 vs. 2.34 ± 1.3; *p* < 0.001) [9]. However, Okano et al. limited cartilage assessments to the medial femoral condyle and did not systematically assess meniscal extrusion or joint effusion [8]. Suwal et al. counted osteophytes rather than grading their severity, used a 2-mm threshold to define meniscal extrusion, and reported all analyses at the patient level without assessing bilateral involvement [9]. Neither study examined the associations between structural and inflammatory US features. Overall, the extent to which US provides additional structural information beyond conventional radiography remains incompletely characterized.

In these studies, bilateral data, where available, were not used for direct within-patient comparison: Okano et al. performed bilateral US assessment in all participants and bilateral radiography in 66 of 84, but reported the analyses at the pooled-knee level, without assessing side-to-side symmetry [8], whereas Suwal et al. reported the analyses at the patient level, without a bilateral assessment [9]. Evaluating both knees in every patient and analyzing each side separately allows direct within-patient comparison, providing insight into whether structural involvement follows a symmetrical pattern or reflects localized mechanical influences.

The aim of this study was to assess musculoskeletal ultrasonography in the bilateral evaluation of patients with knee osteoarthritis, focusing on marginal osteophytes, femoral cartilage thickness, medial JSW, meniscal extrusion, and joint effusion. We hypothesized that ultrasonography would identify marginal osteophytes in knees considered normal or minimally affected on standard weight-bearing radiography. Additionally, we expected that ultrasound-detected meniscal extrusion and joint effusion would be associated with radiographic JSN.

## 2. Materials and Methods

### 2.1. Patient Selection and Criteria

The current observational, cross-sectional study prospectively included 30 consecutive symptomatic patients (60 knees) diagnosed with knee osteoarthritis and evaluated in the Rheumatology Clinic of the Craiova Emergency County Hospital, Romania, between January 2024 and January 2025. The inclusion criteria were: (1) age over 55 years; (2) clinical symptoms suggestive of knee osteoarthritis according to the American College of Rheumatology (ACR) clinical criteria (only patients with persistent symptoms for at least 6 months were included, regardless of radiographic stage); and (3) availability of both weight-bearing radiography and musculoskeletal ultrasound. The exclusion criteria were strictly defined to eliminate confounding factors: (1) previous knee arthroplasty; (2) intra-articular injections within the last 6 months; (3) autoimmune inflammatory joint disease (e.g., rheumatoid arthritis); (4) history of infectious arthritis; and (5) other osteoarticular lesions that could account for joint pain, such as recent fractures or bone tumors. All patients underwent a standardized clinical assessment by a rheumatologist prior to enrollment. No formal functional scores—such as the Western Ontario and McMaster Universities Osteoarthritis Index (WOMAC) or the Visual Analog Scale (VAS)—were collected, as the present study focused on the comparison of imaging findings.

No a priori sample size calculation was performed. Patients were enrolled consecutively over a predefined 12-month period, and all eligible patients presenting during this interval were included. The resulting sample was therefore a consecutive convenience sample from a single tertiary rheumatology center rather than a population-based sample. No sex-based restriction was applied; the female predominance (24/30; 80.0%) reflects the composition of the symptomatic knee OA referral population rather than a predefined selection criterion. The lower age limit of 55 years was selected to favor a cohort predominantly comprising patients with primary knee OA by reducing the likelihood of secondary or post-traumatic disease.

The study protocol was reviewed and approved by the Ethics Committee of the University of Medicine and Pharmacy Craiova (Approval No. 155/30 August 2023). Written informed consent was obtained from all patients before their clinical evaluation, imaging assessment, and inclusion in the study. The research was conducted in accordance with the ethical principles of the Declaration of Helsinki and its later amendments.

### 2.2. Ultrasound Assessment

Ultrasound examinations were performed using a MyLab 25 ultrasound machine (Esaote SpA, Genoa, Italy) equipped with a high-frequency linear transducer (10–18 MHz). All ultrasound assessments were conducted by two experts in musculoskeletal ultrasonography. The examinations were performed independently of the radiographic findings, with both examiners blinded to the radiographic results at the time of ultrasound scanning. Minor interpretative differences were addressed through consensual agreement before recording the final data. The standardized scanning protocol followed the European Alliance of Associations for Rheumatology (EULAR) guidelines [11] with the patient in the supine position. Marginal osteophytes, meniscal extrusion and joint effusion were assessed with the knee in extension, whereas femoral cartilage thickness was measured in maximal flexion (>90°). Joint effusion was defined as an anechoic collection > 4 mm in the suprapatellar recess [12], and osteophytes were assessed at the medial and lateral joint margins, both as presence/absence (binary) and graded on a semi-quantitative scale (0: absent; 1: small; 2: moderate; 3: severe) [8]. Anterior femoral cartilage thickness (mm) was measured with the probe placed transversally in the suprapatellar region at three sites: the medial femoral condyle, the intercondylar notch, and the lateral femoral condyle. Meniscal extrusion was measured on a coronal scan over the medial and lateral joint lines and defined as a displacement of the meniscus > 3 mm beyond the tibial plateau margin [10]. Each knee was classified as showing no extrusion, isolated medial extrusion, isolated lateral extrusion, or bicompartmental extrusion. Analyses of medial compartment parameters were based on the presence of medial extrusion, either isolated or bicompartmental. Ultrasound assessment was performed in grey-scale mode and focused on structural and morphologic features of knee OA. Power Doppler was not systematically included, as the protocol was designed primarily to evaluate structural abnormalities rather than active synovial vascularization, which is generally less prominent in OA than in inflammatory arthropathies. Therefore, synovial/inflammatory involvement was assessed indirectly through grey-scale findings, particularly joint effusion.

### 2.3. Radiographic Assessment

Standard bilateral weight-bearing anteroposterior (AP) radiographs of both knees were obtained for all patients using a fixed acquisition protocol. Patients were examined in the standing position, with both knees fully extended during weight-bearing. Joint space width was measured in millimeters at the narrowest point of the medial and lateral compartments using the digital Picture Archiving and Communication System (PACS) software of Craiova Emergency County Hospital (3Dnet Medical; Biotronics3D Ltd., London, UK; https://www.3dnetmedical.com, accessed on 27 August 2026). Global Kellgren–Lawrence grading [13] was not applied; individual radiographic parameters were assessed separately to allow direct comparison with the corresponding US findings. Marginal osteophytes were assessed separately at the medial and lateral compartments, recorded both as presence/absence and graded on the same semi-quantitative scale used for ultrasound (0: absent; 1: small; 2: moderate; 3: severe). All radiographs were independently evaluated by a single trained investigator who was blinded to the clinical findings and the ultrasonographic results.

### 2.4. Statistical Analysis

The data were analyzed using JMP Student Edition, version 19.0.1 (JMP Statistical Discovery LLC, Cary, NC, USA). Continuous variables are expressed as median (interquartile range, IQR), while categorical and ordinal variables are presented as frequencies and percentages. Analyses were conducted separately for the left and the right knees to account for the non-independence of paired observations within the same patient. Correlations between structural and imaging parameters were analyzed using Spearman’s rank correlation coefficient (*ρ*). Comparisons of continuous variables between groups defined by a binary characteristic were performed using the Mann–Whitney U test. Within-knee comparison of medial versus lateral JSW was performed using the Wilcoxon Signed-Rank test for paired samples. Associations between categorical variables were assessed using Fisher’s exact test. The agreement between ultrasound and radiography in the detection of osteophytes was assessed using a contingency table analysis (McNemar’s test, normal approximation), and the strength of agreement was quantified using Cohen’s kappa coefficient with 95% confidence intervals. Missing data imputation was not required, as complete data were available for all the included subjects. A two-tailed *p*-value < 0.05 was considered statistically significant for all tests. Given the exploratory nature of the analyses, no formal adjustment for multiple comparisons was applied; therefore, the findings should be interpreted in this context.

## 3. Results

The primary objective of this study was to compare the detection yield of ultrasonography with that of standard radiography in the assessment of knee osteoarthritis. The study cohort consisted of 30 patients (24 women, 6 men; median age 69.0 years, range 56–85), each undergoing bilateral clinical and imaging assessment, resulting in a total of 60 evaluated knees. The initial phase of the statistical analysis evaluated the impact of fundamental demographic factors, particularly age and sex, on the severity, structural characteristics, and inflammatory response of joint degeneration.

### 3.1. Patient Profile and Demographic Factors

#### 3.1.1. The Impact of Age on Joint Degeneration

**Figure 1:**
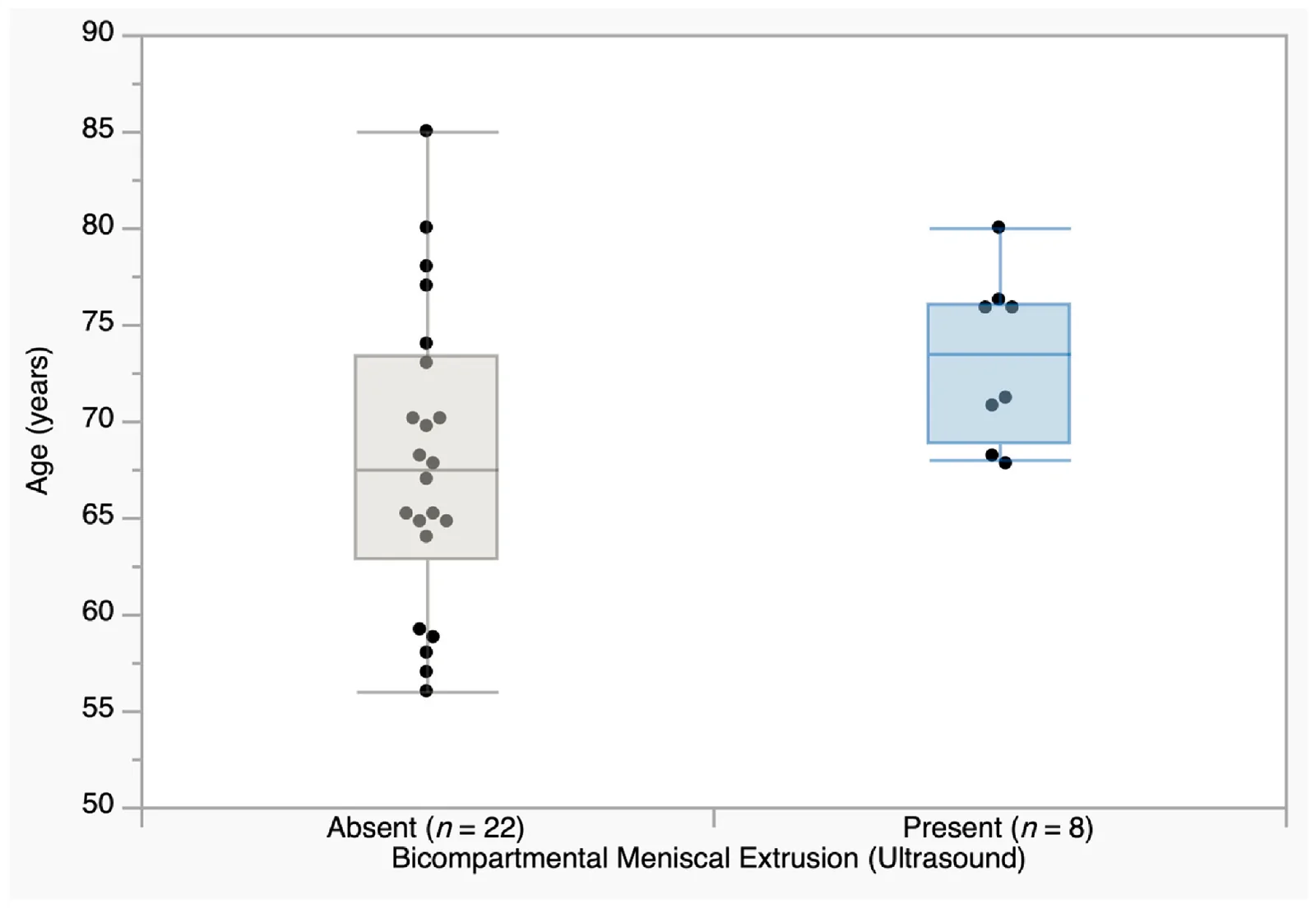
Age distribution of patients with and without bicompartmental meniscal extrusion in the right knee, evaluated by ultrasound (*n* = 30). Box plots show median (horizontal line), interquartile range (box), and 1.5 × IQR whiskers; individual data points are overlaid. Mann–Whitney U test, *p* = 0.048.

To evaluate the impact of age on the risk of meniscal extrusion involving both compartments, patients were divided into two cohorts: those presenting with extrusion of both menisci and those with unilateral or no extrusion. A non-parametric Mann–Whitney U test was performed to compare the age distribution between these two groups. No significant difference was observed for the left knee (*p* = 0.712), whereas a statistically significant result was found for the right knee, where patients with bi-compartmental meniscal extrusion (*n* = 8, median age 73.5 years, IQR 68.6–76.0) were significantly older than those with unilateral or no extrusion (*n* = 22, median age 67.5 years, IQR 62.8–73.3; *p* = 0.048; Figure 1).

Furthermore, Spearman’s rank-order correlation was used to assess the relationship between patient age and the radiographic grading of macroscopic marginal osteophytes (grades 0 to 3). The analysis revealed no statistically significant correlation for either the left knee (*ρ* = −0.278, *p* = 0.136) or the right knee (*ρ* = 0.138, *p* = 0.468). Similarly, to investigate whether age was associated with intra-articular fluid accumulation in osteoarthritic patients, a Mann–Whitney U test was used. Patients were categorized based on the ultrasound-confirmed presence or absence of joint effusion, and their age distributions were compared. The analysis did not reveal a statistically significant difference in age between the two cohorts for either the left knee (*p* = 0.086) or the right knee (*p* = 0.545).

#### 3.1.2. Sex-Based Differences in Imaging Findings

To determine whether sex influences the severity of cartilage degradation, a Mann–Whitney U test was conducted to compare the ultrasound-measured thickness of the medial cartilage between female (*n* = 24) and male (*n* = 6) patients. The analysis did not reveal a statistically significant difference between the two sexes for either the left knee (*p* = 0.286) or the right knee (*p* = 0.483). These findings should be interpreted with caution given the substantial sex imbalance in our cohort, which limits the statistical power for detecting subtle sex-related differences. We performed the same test to evaluate sex-based differences in the context of medial joint space narrowing, measured radiographically in millimeters. Median medial JSW was lower in female patients in both knees (left: 4.59 vs. 5.12 mm; right: 4.00 vs. 4.25 mm), but neither difference reached statistical significance (*p* = 0.436 for the left knee, *p* = 0.640 for the right knee; Table 1).

### 3.2. Distribution and Asymmetry of Structural Joint Degeneration

The distribution of structural joint changes was evaluated, using both ultrasound and radiographic measurements, focusing on compartmental involvement and bilateral symmetry.

#### 3.2.1. Radiographic Asymmetry of JSN

**Figure 2:**
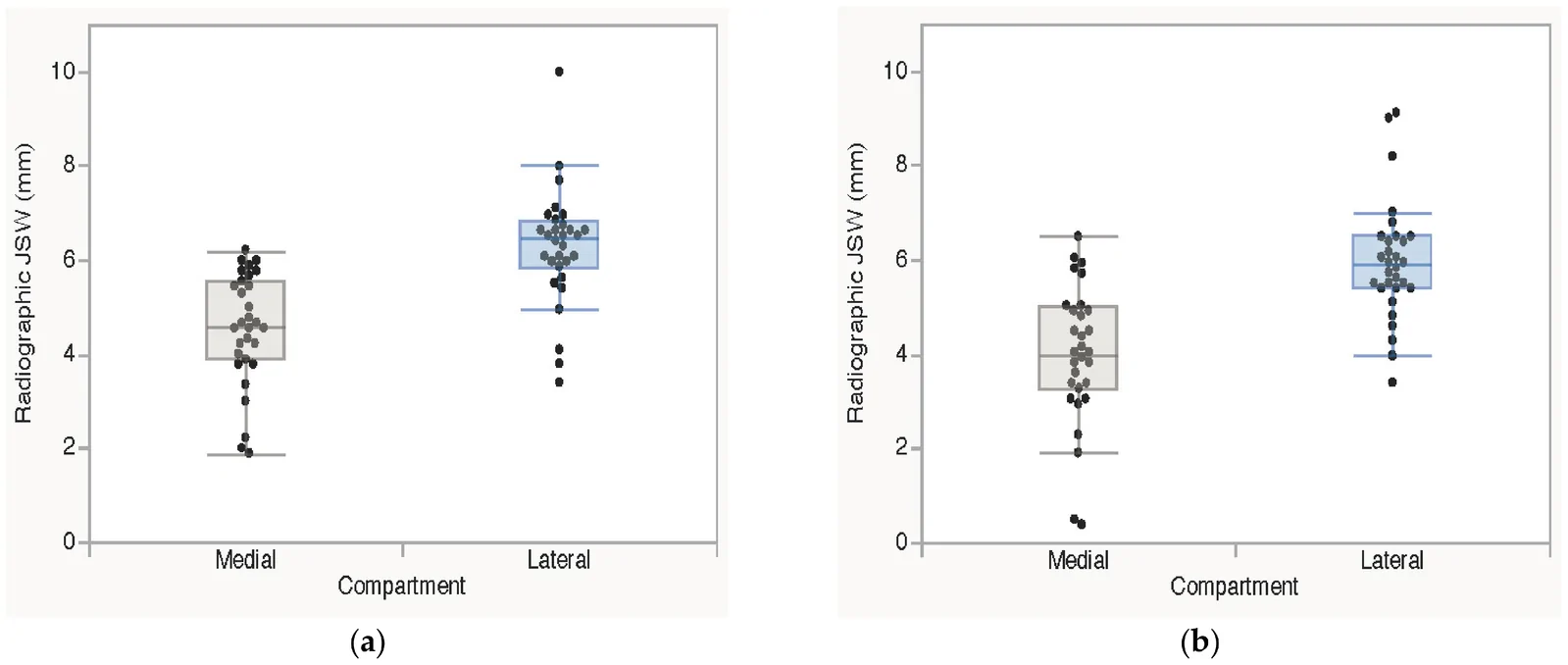
Asymmetrical joint space loss between the medial and lateral compartments evaluated on weight-bearing radiographs. (**a**) Left knee. (**b**) Right knee, exhibiting an even more pronounced asymmetry. Box plots show median (horizontal line), interquartile range (box), and 1.5 × IQR whiskers; individual data points are overlaid. Statistical comparisons were performed using the Wilcoxon Signed-Rank test for paired samples (*p* < 0.0001 for both knees). JSW = joint space width.

To determine whether macroscopic JSN follows an asymmetrical pattern, a Wilcoxon Signed-Rank test for paired samples was used to compare the medial versus lateral JSW, measured radiographically in millimeters, within each knee. The analysis revealed a highly significant difference in both joints. For the left knee, the medial compartment was significantly narrower (median JSW = 4.60 mm) compared to the lateral compartment (median JSW = 6.45 mm, *p* < 0.0001). An even more pronounced asymmetry was observed in the right knee, where the medial joint space narrowed to a median of 4.00 mm, while the lateral compartment measured 5.90 mm (*p* < 0.0001). This asymmetrical pattern is illustrated in Figure 2.

#### 3.2.2. Global Cartilage Degeneration Ultrasonographic Assessment

**Figure 3:**
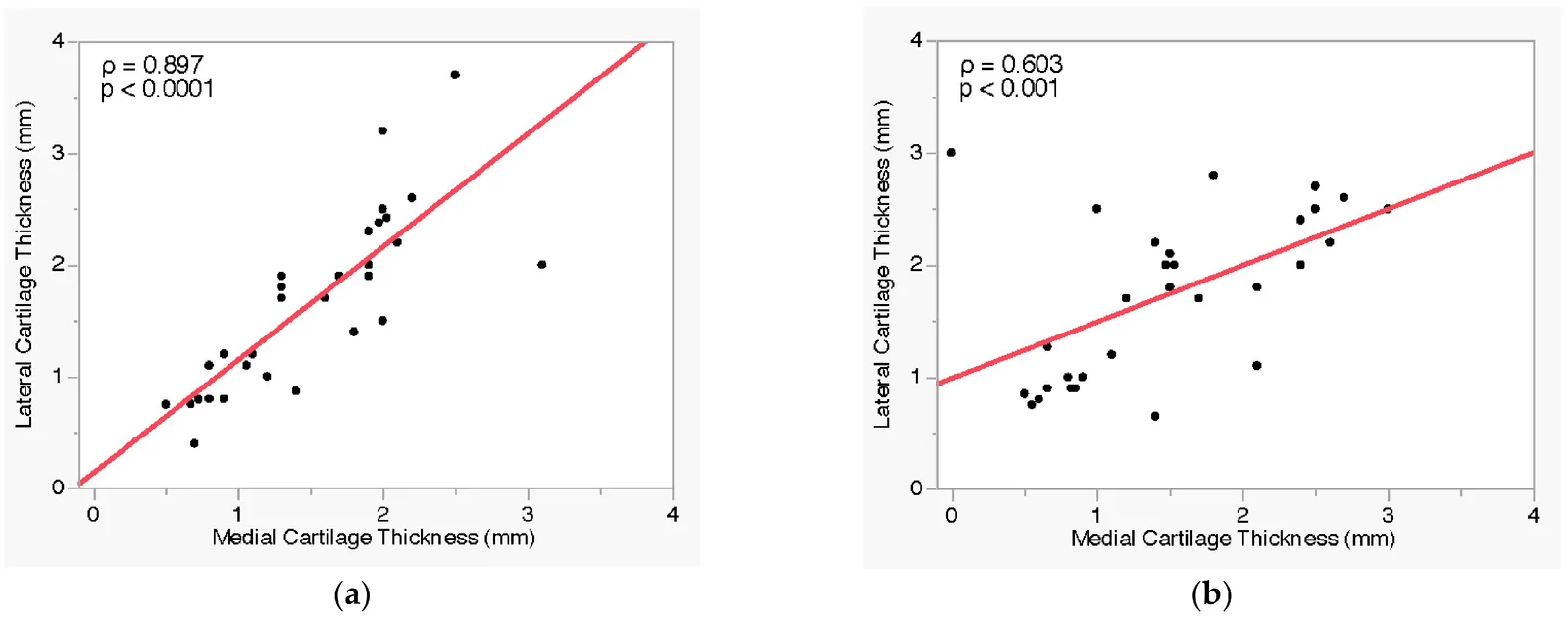
Correlation between ultrasound-measured medial and lateral cartilage thickness. (**a**) Left knee, demonstrating a strong, near-linear positive correlation. (**b**) Right knee, showing a moderate correlation, with several patients presenting advanced medial cartilage loss and a relatively preserved lateral compartment. Each point represents an individual patient (*n* = 30). The solid red line indicates the linear trend. Spearman correlation coefficient: (**a**) *ρ* = 0.897, *p* < 0.0001; (**b**) *ρ* = 0.603, *p* < 0.001.

To investigate whether cartilage degradation is an isolated or a global joint phenomenon, Spearman’s rank-order correlation was performed to assess the relationship between the ultrasound-measured thickness of the medial and lateral articular cartilage. A strong, statistically significant positive correlation was identified between medial and lateral cartilage thickness on ultrasound in both the left knee (*ρ* = 0.897, *p* < 0.0001) and the right knee (*ρ* = 0.603, *p* < 0.001). The notably weaker correlation observed in the right knee was partly attributed to a subset of patients presenting compartmental dissociation, in whom advanced medial cartilage loss coexisted with a relatively preserved lateral compartment. The simultaneous degeneration of the two compartments is illustrated in Figure 3.

**Figure 4:**
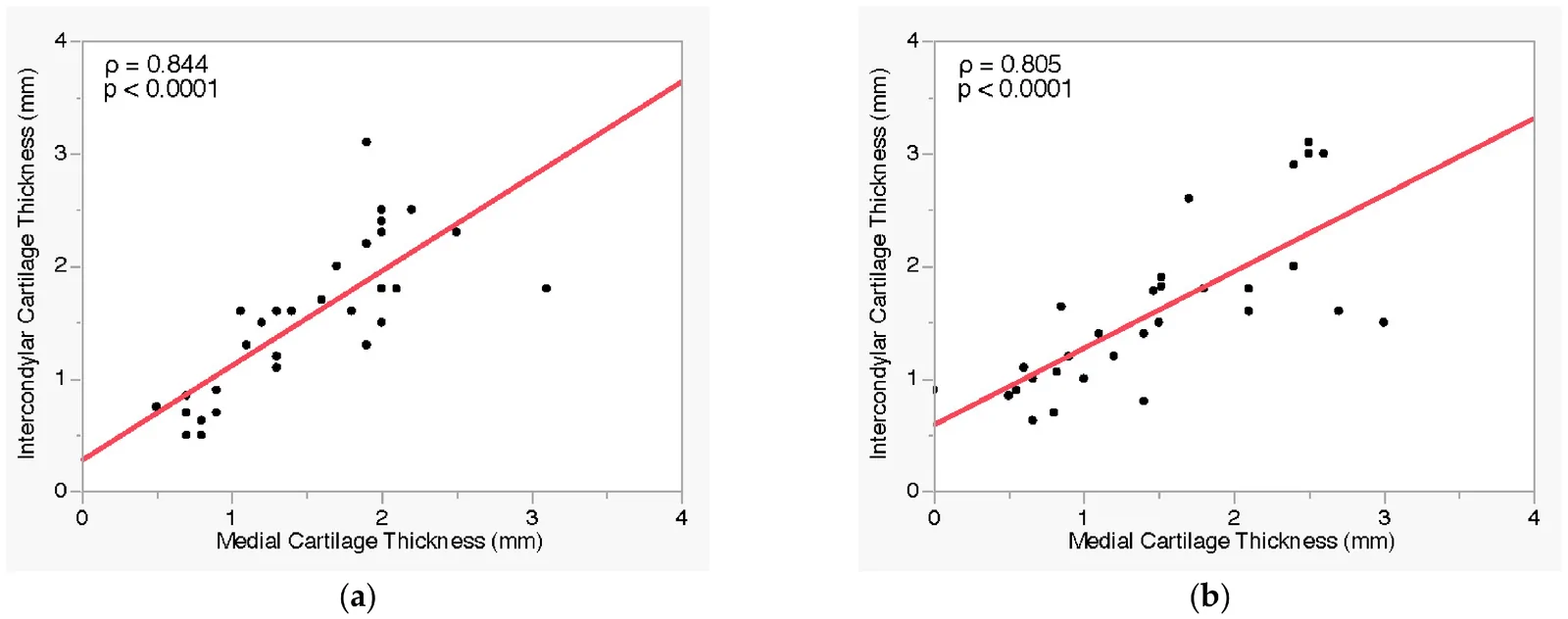
Correlation between ultrasound-measured medial and intercondylar cartilage thickness. (**a**) Left knee. (**b**) Right knee. Each point represents an individual patient (*n* = 30). The solid red line indicates the linear trend. Spearman correlation coefficient: (**a**) *ρ* = 0.844, *p* < 0.0001; (**b**) *ρ* = 0.805, *p* < 0.0001.

Additionally, a strong positive correlation was observed between medial and intercondylar cartilage thickness on ultrasound. This association was highly significant in both the left knee (*ρ* = 0.844, *p* < 0.0001) and the right knee (*ρ* = 0.805, *p* < 0.0001). The corresponding relationships are presented in Figure 4.

#### 3.2.3. Symmetry of Osteophyte Formation

**Figure 5:**
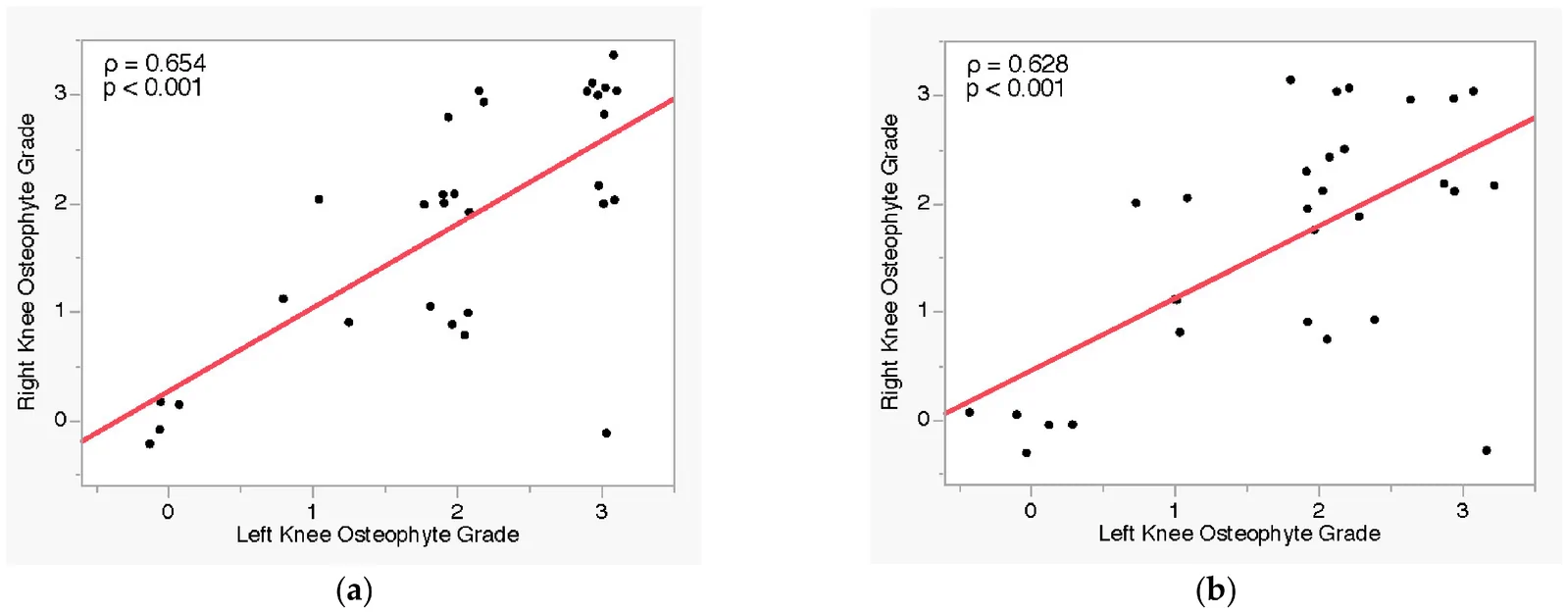
Bilateral symmetry of marginal osteophyte severity assessed by ultrasound. (**a**) Medial compartment, demonstrating a strong positive correlation between the left and right knees. (**b**) Lateral compartment, confirming a similarly strong correlation. Each point represents an individual patient (*n* = 30); points are jittered for visibility due to the discrete ordinal nature of the data; overlapping observations may not be individually distinguishable. The solid red line indicates the linear trend. Spearman correlation coefficient: (**a**) *ρ* = 0.654, *p* < 0.001; (**b**) *ρ* = 0.628, *p* < 0.001.

Furthermore, a topographical synchronization of the compensatory bone response was analyzed using Spearman’s rank-order correlation in order to determine if osteophyte formation develops symmetrically across different joint compartments. A strong, statistically significant positive correlation was identified between the medial compartments of the left and right knees (*ρ* = 0.654, *p* < 0.001), while a similar correlation was also observed for the lateral compartments (*ρ* = 0.628, *p* < 0.001). These bilateral relationships are illustrated in Figure 5.

### 3.3. Diagnostic Concordance Between Ultrasonography and Radiography

In order to evaluate the comparative diagnostic utility of ultrasonography and weight-bearing standard radiography, we analyzed the correlations and concordance rates for the assessment of soft tissue and structural bone changes.

**Figure 6:**
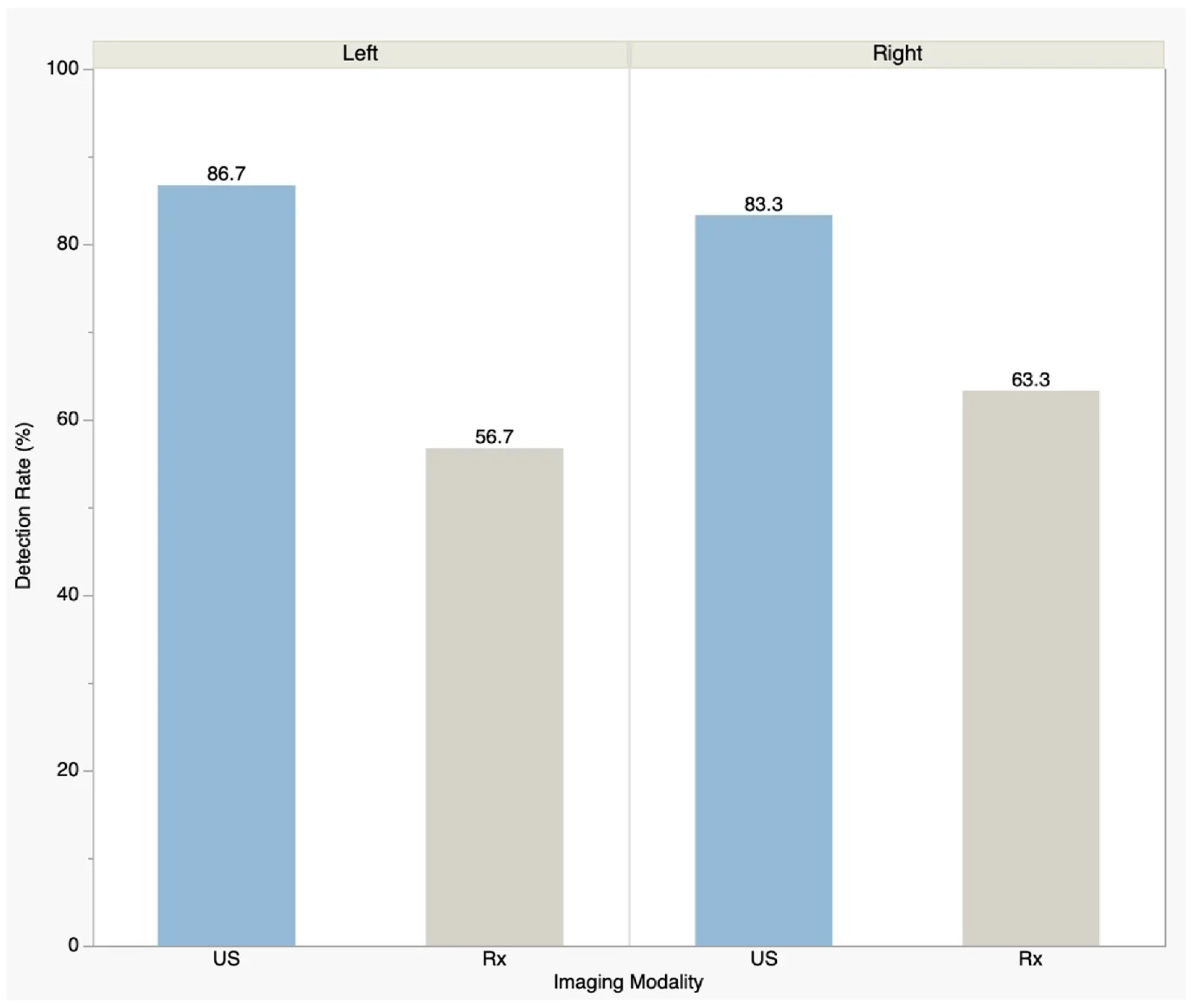
Comparative detection rate of ultrasound (US) and radiography (Rx) for medial marginal osteophytes in the left and right knees. Bars represent the percentage of patients in whom osteophytes were detected by each modality (*n* = 30 per knee). Statistical comparison was performed using McNemar’s test for paired nominal data (*p* = 0.003 for the left knee; *p* = 0.014 for the right knee).

First, the relationship between the direct ultrasonographic measurement of cartilage thickness and the radiographic JSW was investigated using Spearman’s rank-order correlation coefficient. The statistical analysis revealed no significant correlation between the two measurements: a weak, non-significant negative correlation was observed in the left knee (*ρ* = −0.266, *p* = 0.156) and no correlation in the right knee (*ρ* = 0.092, *p* = 0.628). Second, the detection rates of the two methods for medial marginal osteophytes were compared. The ordinal severity grades were converted into a binary variable (presence versus absence), and a McNemar test for paired nominal data was applied to evaluate the diagnostic concordance within the same cohort. Ultrasound identified the presence of osteophytes in a substantially higher proportion of cases that were classified as negative (grade 0) on standard radiography. In the left knee, US detected osteophytes in 86.7% of patients (26/30) compared to 56.7% (17/30) by radiography, with 9 cases identified exclusively by US (*p* = 0.003). A similar pattern was observed in the right knee, where US detected osteophytes in 83.3% of patients (25/30) compared to 63.3% (19/30) by radiography, with 6 cases identified exclusively by US (*p* = 0.014). Notably, no osteophytes were detected by radiography that were missed by ultrasound. Agreement between the two modalities was κ = 0.34 (95% CI 0.06–0.61; observed agreement 70.0%) in the left knee and κ = 0.51 (95% CI 0.21–0.82; observed agreement 80.0%) in the right knee. The detection rates of two imaging modalities are illustrated in Figure 6.

In absolute terms, radiography failed to identify marginal osteophytes in 9 of the 26 US-positive left knees (34.6%) and in 6 of the 25 US-positive right knees (24.0%). All 15 discordant knees showed the same unidirectional pattern (US-positive/radiography-negative), and the same direction of discordance was observed in the left and right knee analyses, which were conducted separately.

### 3.4. Structural Associations of Ultrasonographic Markers

#### 3.4.1. Meniscal Extrusion and Structural Findings

Meniscal extrusion was absent in 6 left and 4 right knees, isolated medial in 16 left and 17 right knees, isolated lateral extrusion in 1 left and 1 right knee, and bicompartmental in 7 left and 8 right knees. The impact of meniscal extrusion on cartilage morphology and joint space narrowing was evaluated using nonparametric comparative analyses. Medial meniscal extrusion was not associated with a statistically significant reduction in medial cartilage thickness, either in the left knee (*p* = 0.768) or the right knee (*p* = 0.558). Similarly, the analysis of articular cartilage asymmetry—quantified as the difference between lateral and medial thicknesses—showed no significant variation when comparing patients with medial meniscal extrusion to those without (left knee *p* = 0.523; right knee *p* = 0.330).

**Figure 7:**
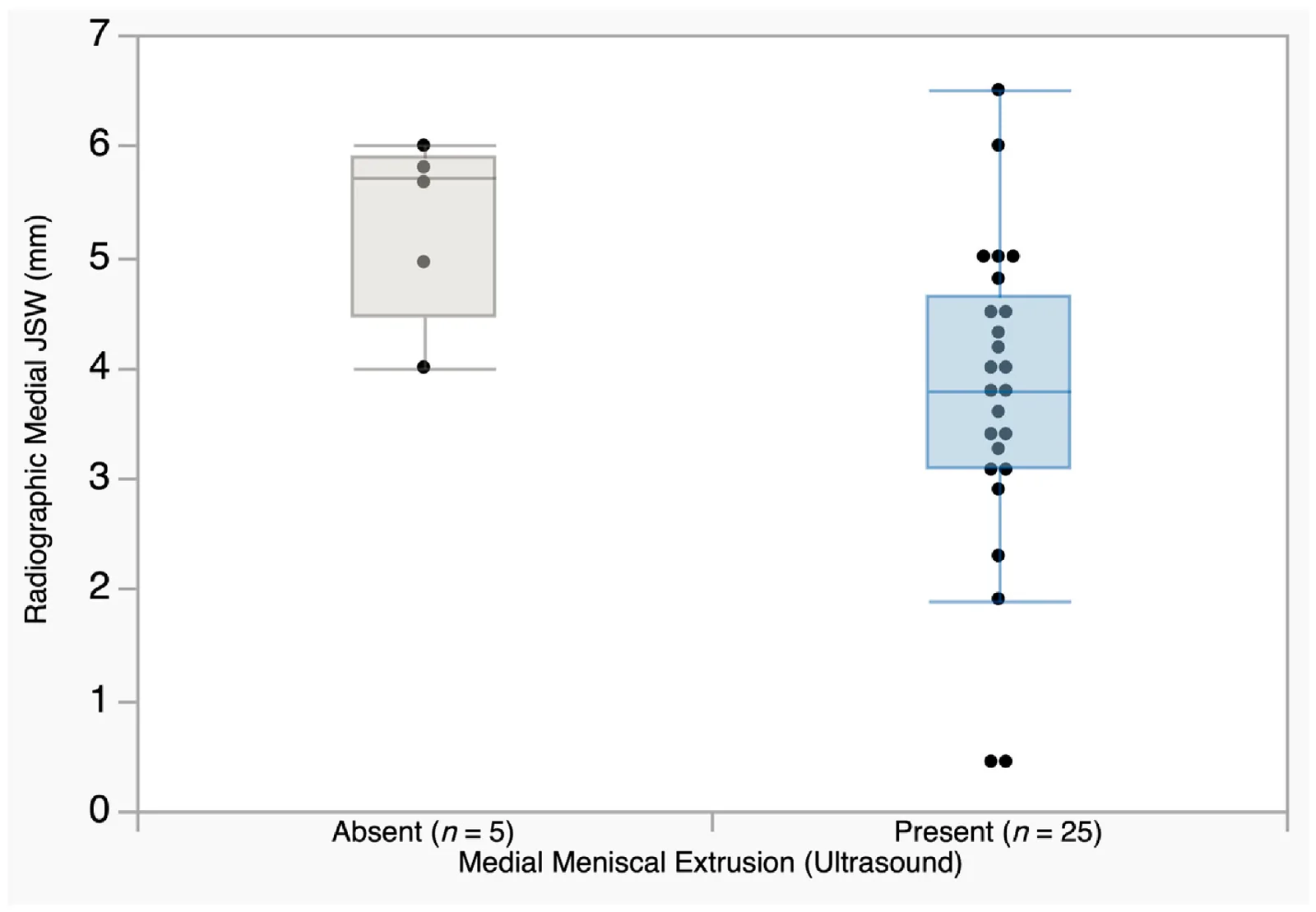
Comparison of radiographic medial JSW between patients with and without ultrasound-detected medial meniscal extrusion in the right knee. Patients with medial meniscal extrusion (*n* = 25) showed significantly narrower JSW compared to those without extrusion (*n* = 5). Box plots show median (horizontal line), interquartile range (box), and 1.5 × IQR whiskers; individual data points are overlaid. Statistical comparison was performed using the Mann–Whitney U test (*p* = 0.024). JSW = joint space width.

Furthermore, a significant relationship was identified when assessing the impact of medial meniscal extrusion on radiographic findings. The presence of medial meniscal extrusion was associated with a statistically significant narrowing of the medial joint space on weight-bearing radiographs in the right knee (*p* = 0.024; Figure 7), while in the left knee, the same direction of association did not reach statistical significance (*p* = 0.073).

The median JSW differed by 1.94 mm between knees with and without medial meniscal extrusion, corresponding to approximately one-third of the median JSW in knees without extrusion. Given the small number of knees without extrusion (*n* = 5), this estimate should be interpreted with caution.

#### 3.4.2. Osteophyte Formation and Structural Associations

**Figure 8:**
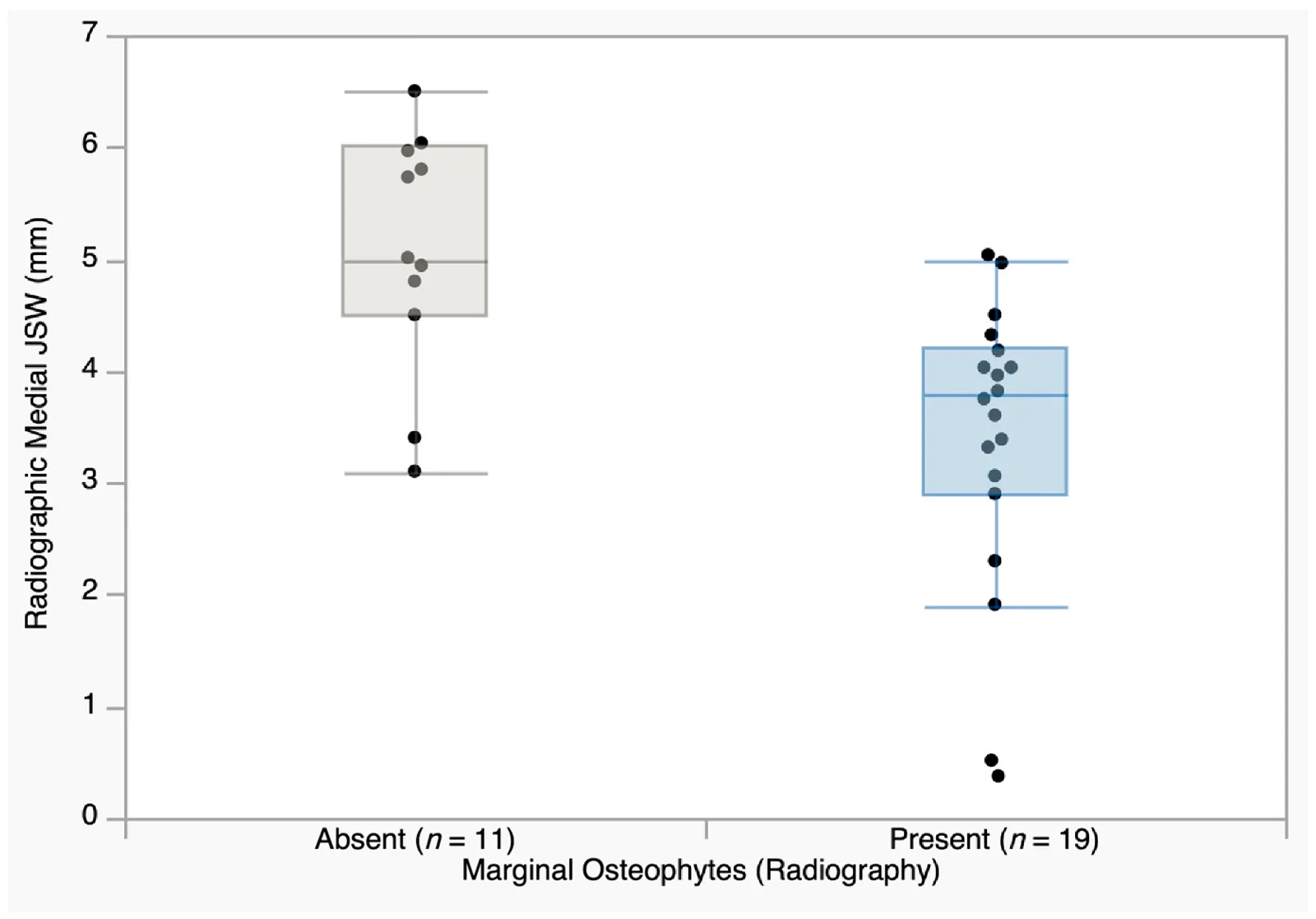
Comparison of radiographic medial JSW between patients with and without radiographically detected marginal osteophytes in the right knee. Patients with radiographic osteophytes (*n* = 19) showed significantly narrower JSW (median 3.79 mm) compared to those without (*n* = 11; median 5.00 mm). Box plots show median (horizontal line), interquartile range (box), and 1.5 × IQR whiskers; individual data points are overlaid. Statistical comparison was performed using the Mann–Whitney U test (*p* = 0.002). JSW = joint space width.

The relationship between osteophyte severity and medial cartilage thickness was investigated to assess patterns of joint degeneration. Spearman’s rank-order correlation did not reveal a statistically significant relationship between the ultrasound-measured grade of medial osteophytes and medial cartilage thickness, either in the left knee (*ρ* = −0.027, *p* = 0.887) or the right knee (*ρ* = −0.261, *p* = 0.164). Further analysis evaluated whether the formation of radiographically visible marginal osteophytes corresponds to a reduction in the joint space. A Mann–Whitney U test was performed to compare the medial JSW (mm) between patients grouped by the presence or absence of osteophytes on standard radiographs. No significant difference in medial JSW was observed in the left knee (*p* = 0.676), with median values of 4.70 mm in patients with osteophytes and 4.55 mm in those without osteophytes. In contrast, a statistically significant reduction in JSW was identified in the right knee (*p* = 0.002), where patients with osteophytes presented a median JSW of 3.79 mm compared to 5.00 mm in those without (Figure 8).

The relationship between meniscal extrusion and osteophyte severity was also assessed using a Mann–Whitney U test. Patients were stratified into two cohorts (intact vs. extruded menisci), and their medial osteophyte severity grade (0–3) was compared. In the left knee, medial osteophyte grade did not differ between knees with or without meniscal extrusion (median grade 2 in both groups; *p* = 0.658). In the right knee, medial osteophyte grade was higher in knees with meniscal extrusion (median grade 2) than in those without (median grade 0), but the difference did not reach statistical significance (*p* = 0.125).

### 3.5. Inflammatory Involvement and Ultrasonographic Markers Associated with JSN

#### 3.5.1. Joint Effusion and Structural Abnormalities

**Figure 9:**
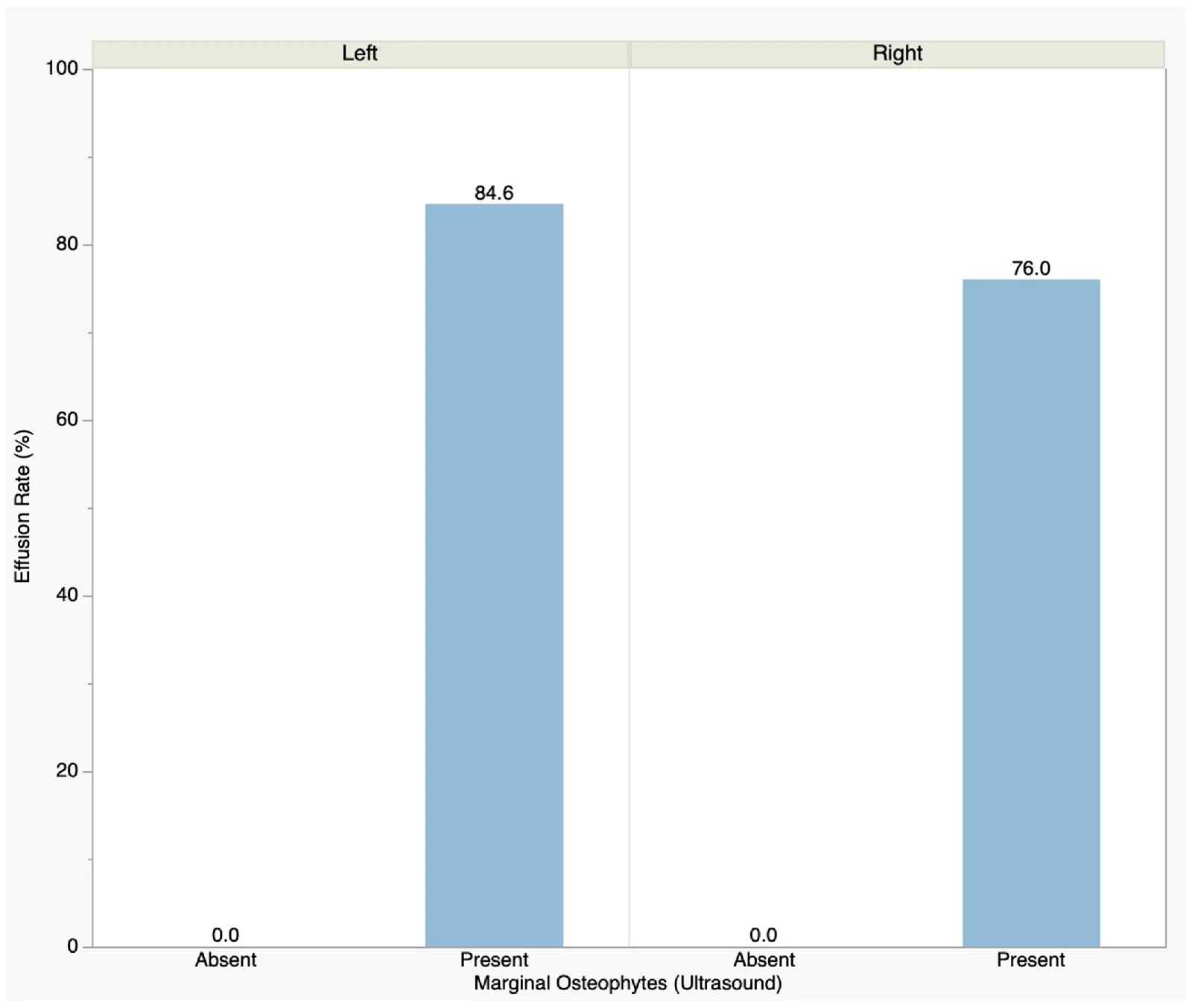
Frequency of joint effusion in patients with versus without ultrasound-detected marginal osteophytes in the left and right knees. Effusion was present in 84.6% (22/26) of left knees with osteophytes versus 0% (0/4) of those without and in 76.0% (19/25) versus 0% (0/5) in the right knee. Statistical comparisons were performed using Fisher’s Exact Test (*p* = 0.003 for both knees). Notably, no case of joint effusion occurred in the absence of marginal osteophytes.

A categorical analysis using Fisher’s exact test was performed to evaluate the association between ultrasound-detected marginal osteophytes and joint effusion. The analysis revealed a highly significant positive association in both knees. In the left knee, effusion was present in 84.6% of patients with osteophytes (22/26) compared to 0% of those without (0/4; *p* = 0.003). A similar pattern was observed in the right knee, where effusion was present in 76.0% of patients with osteophytes (19/25) versus 0% of those without (0/5; *p* = 0.003). Notably, no case of joint effusion was observed in the absence of marginal osteophytes. This relationship is illustrated in Figure 9.

**Figure 10:**
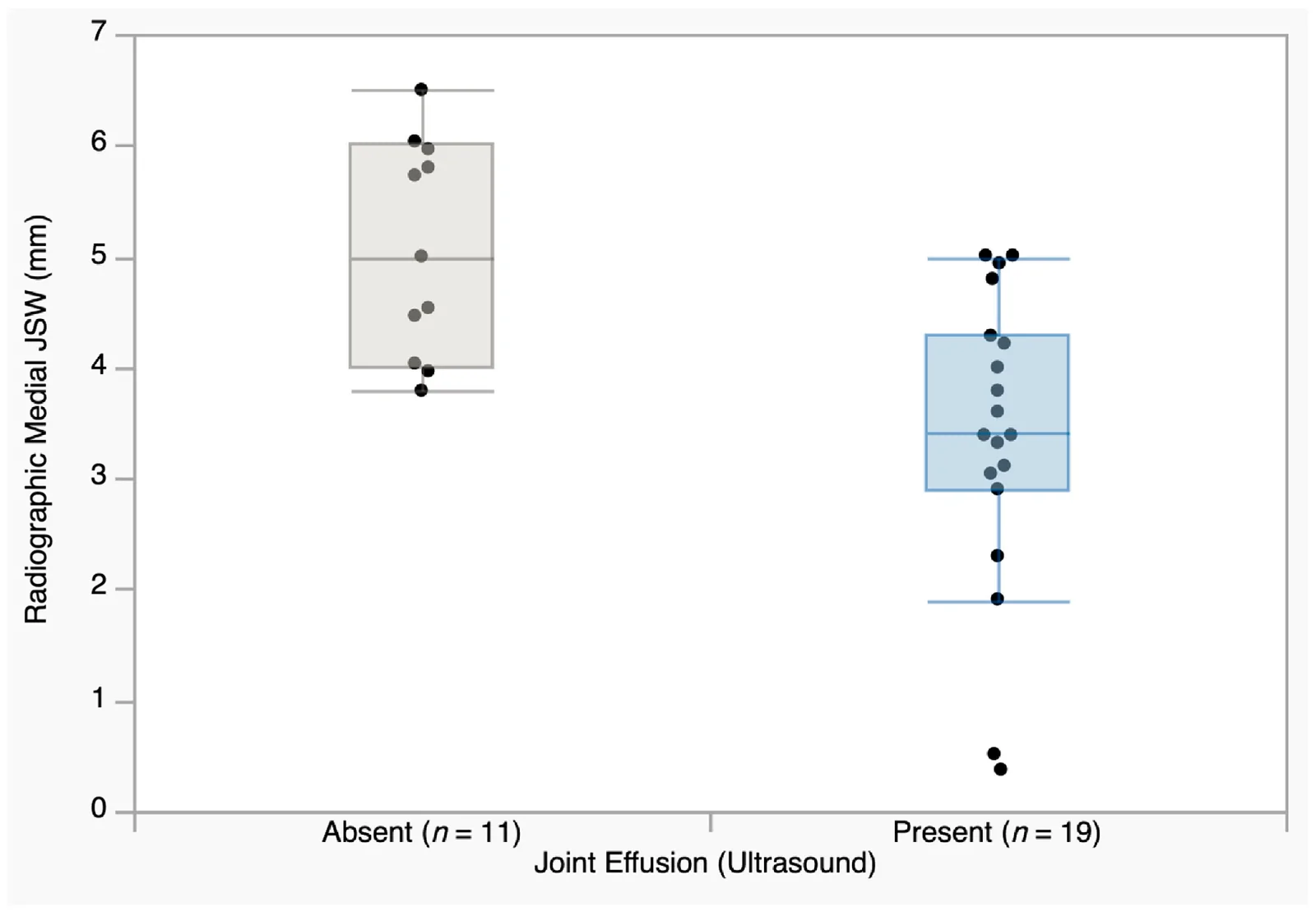
Comparison of radiographic medial joint space width (JSW) between patients with and without ultrasound-detected joint effusion in the right knee. Patients with joint effusion (*n* = 19) showed significantly narrower JSW (median 3.40 mm) compared to those without (*n* = 11; median 5.00 mm). Box plots show median (horizontal line), interquartile range (box), and 1.5 × IQR whiskers; individual data points are overlaid. Statistical comparison was performed using the Mann–Whitney U test (*p* = 0.001). JSW = joint space width.

The relationship between ultrasound-detected joint effusion and radiographic JSW was evaluated using the Mann–Whitney U test. In the right knee, patients with joint effusion (*n* = 19) showed a significantly narrower medial JSW (median 3.40 mm) compared to those without effusion (*n* = 11; median 5.00 mm; *p* = 0.001; Figure 10). In the left knee, a similar trend was observed, but did not reach statistical significance (median 4.56 mm with effusion vs. 5.15 mm without; *p* = 0.111).

#### 3.5.2. Comparative Analysis of Ultrasonographic Markers Associated with JSN

To determine the relative contribution of distinct ultrasonographic findings to the radiographic medial joint space width, Mann–Whitney U tests were used to compare JSW between patients with and without each marker (meniscal extrusion, medial osteophytes, and joint effusion). The analysis revealed a different pattern of associations between the two knees, as detailed in Table 2. In the left knee, none of the three markers reached statistical significance, although meniscal extrusion showed the strongest numerical association with medial JSN (∆ JSW = 1.22 mm; *p* = 0.073), followed by joint effusion (*p* = 0.111) and medial osteophytes (*p* = 0.879). In contrast, in the right knee, all three markers were significantly associated with JSW narrowing, with joint effusion showing the lowest *p*-value (∆ JSW = 1.60 mm; *p* = 0.001), followed by medial osteophytes (∆ JSW = 1.94 mm; *p* = 0.009) and meniscal extrusion (∆ JSW = 1.94 mm; *p* = 0.024). In the right knee, medial meniscal extrusion and medial osteophytes demonstrated substantial concordance (28/30 knees; 24 positive for both findings), resulting in identical median JSW values and comparable ∆ JSW. These associations should therefore not be interpreted as independent contributions.

## 4. Discussion

The present study evaluates the added value of musculoskeletal ultrasonography in knee osteoarthritis by assessing structural and inflammatory abnormalities that extend beyond the information provided by standard weight-bearing radiography. We first examined the contribution of demographic factors. Osteoarthritis has traditionally been conceptualized as a degenerative condition closely linked to aging; in our cohort, however, patient age did not correlate with the radiographic grade of marginal osteophytosis (*p* = 0.136 for the left knee; *p* = 0.468 for the right knee). This finding is consistent with previous evidence suggesting that osteophyte formation is a localized, compensatory adaptive response to mechanical stress, rather than simply reflecting aging-related processes [14].

Regarding meniscal extrusion, a significant age-related association was observed only in the right knee, where patients with meniscal extrusion were older than those without extrusion (*p* = 0.048). In contrast, no similar association was found in the left knee (*p* = 0.712), suggesting that meniscal displacement may be influenced not only by cumulative mechanical wear, but also by local biomechanical factors such as malalignment or unequal loading [15,16]. This interpretation is consistent with previous evidence linking meniscal extrusion to joint space narrowing and structural progression in knee OA [17].

A different pattern was observed for joint effusion. There was no significant age difference between patients with and without joint effusion (*p* = 0.086 for the left knee; *p* = 0.545 for the right knee). This supports the current view of osteoarthritis as an active whole-joint disease, in which synovial changes are closely related to both structural progression and clinical manifestations [18,19]. However, as power Doppler ultrasound and synovial hypertrophy were not systematically evaluated, the inflammatory nature of the effusion could not be determined.

While epidemiological data recognize female sex as a major risk factor for the incidence and accelerated progression of knee OA [20], our results did not show a statistically significant difference between female and male patients regarding medial cartilage thickness (*p* = 0.286 left; *p* = 0.483 right) or medial JSW (*p* = 0.436 left; *p* = 0.640 right).

### 4.1. Structural Pattern and Imaging Concordance of Knee Osteoarthritis

Following these findings, we extended the analysis to evaluate the pattern of structural degeneration across the joint. Although knee osteoarthritis has been described as a focal “wear-and-tear” pathology, localized to areas of high biomechanical stress, our data support the concept that cartilage degeneration in knee osteoarthritis may involve multiple joint regions in a coordinated, global process, rather than remaining limited to a single compartment. This is evidenced by the strong correlations observed between medial and lateral cartilage thickness (left knee: ρ = 0.897, *p* < 0.0001; right knee: ρ = 0.603, *p* < 0.001), indicating that cartilage loss in the primary load-bearing region coexists with structural changes in the remaining compartments. While the strong correlation observed in the left knee supports the concept of cartilage degeneration as a global joint phenomenon, the weaker correlation in the right knee was driven by a minority of patients with marked compartmental dissociation, in whom complete medial cartilage loss coexisted with a relatively preserved lateral compartment. These cases illustrate how localized biomechanical factors may override the global degenerative pattern in individual patients, although lower-limb alignment was not quantitatively assessed in the present study. Moreover, a strong correlation between the degeneration of the medial compartment and the intercondylar notch (*ρ* = 0.844 and *ρ* = 0.805 for the left and right knees, respectively; both *p* < 0.0001) was observed. The concurrent involvement of the intercondylar area—a region with lower mechanical loading—supports the idea that disease progression is not solely determined by mechanical wear. Instead, it suggests that mechanical damage and a broader intra-articular inflammatory environment may coexist, together contributing to more diffuse structural involvement [3,19]. Furthermore, these findings emphasize the usefulness of musculoskeletal ultrasonography in detecting structural changes across different joint areas [12].

Despite this global degenerative pattern, radiographic assessment revealed a marked compartmental asymmetry, with significantly greater narrowing of the medial compartment than of the lateral compartment in both knees (*p* < 0.0001). This apparent discrepancy emphasizes the distinction between diffuse cartilage degradation and compartment-specific mechanical failure. Previous studies have shown that medial meniscal extrusion is associated with multiple markers of disease severity, including cartilage damage, osteophyte formation, varus alignment, and progression over time [16]. Moreover, recent systematic reviews and cohort studies have confirmed that meniscal extrusion is not only a marker but also a predictor of structural progression [10,17].

In addition to this intra-articular asymmetry, our analysis revealed a bilateral pattern of structural involvement. A strong correlation was observed between the left and right knees regarding the severity of medial marginal osteophytosis (*ρ* = 0.654, *p* < 0.001), along with a similar correlation in the lateral compartments (*ρ* = 0.628, *p* < 0.001). This bilateral pattern suggests that knee osteoarthritis should not be interpreted exclusively as an isolated joint disorder, but rather as an interdependent process. When structural failure and malalignment develop in one joint, the resulting compensatory gait alterations may modify loading patterns and increase mechanical stress on the contralateral knee, potentially contributing to similar—though not identical—osteophytic response across both knees [15,21]. Neither of the two previous comparative studies assessed bilateral symmetry, although Okano et al. examined both knees in all participants [8]. The moderate-to-strong left–right correlations observed here therefore add information that feature-specific comparison alone does not provide.

A primary objective of this study was to systematically compare the detection rates of ultrasonography and standard weight-bearing radiography for individual osteoarthritic features. One of the main findings of our analysis indicated that ultrasonography detected marginal osteophytes in knees classified as negative for osteophytes on standard radiography, with a statistically significant discordance between the two methods in both the left (*p* = 0.003) and right knee (*p* = 0.014). This finding is consistent with previous comparative studies reporting a higher detection rate of ultrasonography than conventional radiography for osteophyte detection in knee osteoarthritis [8,9]. The higher detection rate is likely related to the direct visualization of superficial bone margins by ultrasound, whereas radiography may underestimate early marginal remodeling due to its two-dimensional projection and anatomical limitations [6,7]. Early marginal bone remodeling may become identifiable on ultrasound before it reaches the threshold of radiographic visibility.

Our detection rates (US 86.7% and 83.3% vs. radiography 56.7% and 63.3%) are directionally consistent with those reported by Okano et al., who identified US osteophytes in 20 of 46 medially and 26 of 66 laterally radiography-negative knees [8], and by Suwal et al., who reported a higher mean osteophyte count on US than on radiography (4.04 vs. 2.34; *p* < 0.001) [9]. The magnitude of discordance was greater in our cohort, which may reflect several methodological and population differences. First, our patients were recruited from a rheumatology clinic and were all symptomatic and older than 55 years, whereas Suwal et al. included patients aged 18 years and older presenting to a radiology department [9], a population in which early structural changes may have been less prevalent. In contrast, the cohort studied by Okano et al. had a mean age of 68.7 years and was recruited from a rheumatology outpatient clinic, making it more comparable to our population [8]. Second, the outcome definitions were not directly comparable: in our study, osteophytes were recorded as present or absent at the medial and lateral joint margins, whereas Suwal et al. counted individual osteophytes along the joint line, and Okano et al. graded them by size at the femoral condyles [8,9]. Third, ultrasonography in the present study was performed using a 10–18 MHz transducer, compared with a 4–13 MHz probe in Okano et al. [8], representing a difference in imaging specifications that may have influenced the detection of small marginal changes. The corresponding kappa values (0.34 and 0.51) indicate limited agreement between the two modalities, further supporting their complementary rather than interchangeable nature. The unidirectional nature of the discordance, observed consistently in both separately conducted analyses, is compatible with a difference in detection between the two modalities rather than random measurement variability. Clinically, these findings indicate that a negative radiographic result does not necessarily exclude marginal osteophytes detectable by ultrasound in patients with symptomatic knee OA.

A notable finding is the absence of a significant correlation between ultrasonographic medial cartilage thickness and radiographic medial joint space width (left knee: *ρ* = −0.266, *p* = 0.156; right knee: *ρ* = 0.092, *p* = 0.628), indicating that the two methods are not interchangeable, as they capture different aspects of joint pathology. This is consistent with previous evidence that radiographic JSW does not exclusively reflect cartilage status in knee osteoarthritis [4,22]. Okano et al. reported a strong correlation between US cartilage grade and radiographic tibiofemoral narrowing (*ρ* = 0.714) [8], whereas we found no significant correlation between US cartilage thickness and radiographic medial JSW. They compared a semi-quantitative cartilage damage grade with a feature-specific radiographic narrowing grade and assessed cartilage through the medial parapatellar acoustic window, whereas we used continuous thickness measurements obtained in the suprapatellar region at three sites and compared them with JSW measured in millimeters. These differences in measurement approach and anatomical sampling limit the direct comparability of the two findings. Thus, ultrasonography and radiography provide complementary structural information.

### 4.2. Meniscal Extrusion as a Structural Marker

Extending the previous imaging findings, we further assessed the role of meniscal extrusion as a compartment-specific marker of structural joint damage. One of the most clinically relevant findings was the association between ultrasound-detected meniscal extrusion and radiographic medial JSN, which reached statistical significance in the right knee (*p* = 0.024) and showed a consistent non-significant trend in the left knee (*p* = 0.073). This association is consistent with previous studies showing that radiographic JSW is not determined by cartilage thickness alone, but is also substantially influenced by meniscal position and extrusion [5,22].

Recent evidence has validated ultrasonography as a reliable method for assessing meniscal extrusion [23], also indicating that ultrasound may predict the development of radiographic knee OA [24]. The relationship between medial meniscal extrusion and ultrasonographic medial cartilage thickness was also assessed, with no statistically significant difference in either knee (left: *p* = 0.768; right: *p* = 0.558). Although these differences did not reach statistical significance—primarily due to the small number of knees without meniscal extrusion—the direction of the observed difference is consistent with prior longitudinal imaging evidence showing an association between medial meniscal extrusion and subsequent medial compartment cartilage loss [25,26]. A similar directional pattern was observed for medial–lateral cartilage asymmetry, with knees presenting meniscal extrusion showing a numerical trend toward greater asymmetry compared to those without extrusion. This trend is biomechanically plausible, as progressive meniscal extrusion has been shown to alter tibiofemoral load distribution and increase focal compartmental stress [27]. Suwal et al. reported lower medial femoral cartilage thickness in patients with extrusion (0.50 vs. 0.83 mm; *p* = 0.003) [9], whereas we found no significant difference, having assessed a different cartilage region under a different degree of knee flexion. They also detected meniscal extrusion in 15% of patients using a 2-mm threshold [9], whereas medial extrusion was present in 76.7% of left and 83.3% of right knees in our cohort despite the stricter 3-mm threshold. Because the higher threshold would be expected to reduce the proportion classified as having extrusion, the substantially higher prevalence in our cohort is more likely related to differences in the study population, including the symptomatic, older participants recruited from a rheumatology clinic. Notably, Okano et al. attributed part of the discordance between cartilage status and radiographic narrowing to meniscal subluxation [8], and our finding of an association between medial meniscal extrusion and medial JSN in the right knee (*p* = 0.024) is consistent with that interpretation.

Following the analysis of meniscal extrusion and compartmental JSN, we further evaluated whether osteophyte formation reflected cartilage loss in a proportional manner. In our cohort, we found no statistically significant inverse correlation between the severity grade of medial osteophytes and medial cartilage thickness (*p* = 0.887 for the left knee; *p* = 0.164 for the right knee). This finding suggests that, although osteophyte formation and cartilage degeneration are both central features of knee osteoarthritis, they do not necessarily progress in a strictly synchronized or linear manner. Instead, it indicates that osteophytosis develops as an independent biological and biomechanical process, which may not directly parallel ultrasound-measured cartilage thinning [14]. This interpretation was further supported by the radiographic findings. The presence of osteophytes visible on X-ray was not associated with medial JSN in the left knee (*p* = 0.676), where median JSW values were similar between knees with and without osteophytes (4.70 mm versus 4.55 mm). By contrast, a strong association was observed in the right knee (*p* = 0.002), where radiographically visible osteophytes were accompanied by marked medial JSN (3.79 mm versus 5.00 mm). This unilateral pattern suggests that osteophytes may become more closely linked to radiographic compartmental collapse when local mechanical failure is more advanced, rather than reflecting cartilage loss uniformly across both knees. The right-sided association may therefore reflect a more advanced local biomechanical phenotype, in which medial compartment narrowing, altered load distribution, and marginal bone remodeling coexist, although this was not formally tested. A similar directional pattern was observed for the relationship between meniscal extrusion and osteophyte severity. Although the association did not reach conventional statistical significance, in the right knee, osteophyte severity was higher in knees with meniscal extrusion than in those without (*p* = 0.125). This suggests that meniscal displacement may be part of a local biomechanical cycle that favors marginal bone remodeling [28,29]. Consequently, osteophytosis may be interpreted as part of a local adaptive remodeling response associated with soft-tissue dysfunction and altered joint mechanics, supporting the value of early ultrasound assessment for identifying potentially modifiable structural abnormalities.

### 4.3. Effusion and Mechano-Inflammatory Involvement

Building on the assessment of meniscal extrusion and osteophytosis as markers of local biomechanical stress, we further evaluated whether these structural abnormalities were associated with ultrasound-detected joint effusion. In our cohort, the presence of osteophytes was significantly associated with joint effusion in both knees (left: *p* = 0.003; right: *p* = 0.003). Notably, effusion was observed only in knees with osteophytosis, whereas osteophyte-negative knees showed no detectable fluid accumulation. This finding suggests that joint effusion may coexist with marginal bone remodeling as part of a broader structural involvement in knee OA. While power Doppler assessment and synovial hypertrophy evaluation were not systematically performed, the presence of joint effusion may still be interpreted as a relevant grey-scale indicator of synovial involvement associated with structural remodeling in knee OA. This association should not be interpreted as evidence of direct causality, but rather as an indicator that osteophytosis may identify a mechanically active intra-articular state in which effusion-based synovial involvement is more likely to occur. Marginal osteophytes may contribute to local mechanical irritation while also reflecting a more advanced structural phenotype. This interpretation is consistent with current concepts recognizing synovitis and effusion as integral components of osteoarthritis pathophysiology rather than incidental findings [30]. Neither previous study examined joint effusion in relation to structural findings [8,9]. The observation that effusion occurred exclusively in knees with US-detected osteophytes therefore adds a dimension that feature-specific structural comparison does not capture.

The relationship between medial JSW and joint effusion further supported a link between compartmental structural damage and intra-articular fluid accumulation. In the right knee, patients with joint effusion had significantly narrower medial JSW than those without effusion (*p* = 0.001), whereas no significant difference was found in the left knee (*p* = 0.111). This asymmetry suggests that effusion may become more evident when compartmental mechanical failure is more advanced, although the two knees were not directly compared. The current observation supports the interpretation of effusion as a marker of mechano-inflammatory involvement rather than an isolated finding. Cartilage degradation, meniscal abnormalities, marginal bone remodeling, and subchondral stress may all contribute to synovial stimulation and fluid accumulation. Longitudinal studies have similarly linked effusion–synovitis with structural deterioration in knee osteoarthritis, although this relationship is likely bidirectional rather than purely causal [31]. The comparative analysis of ultrasound markers further refined this interpretation. In the left knee, meniscal extrusion showed the strongest numerical association with medial JSN (*p* = 0.073), although no marker reached statistical significance. By contrast, in the right knee, all three ultrasound markers were significantly associated with JSW narrowing, with effusion showing the lowest *p*-value (*p* = 0.001), followed by medial osteophytes (*p* = 0.009) and meniscal extrusion (*p* = 0.024). This pattern suggests a possible stage-dependent hierarchy of ultrasound findings. Meniscal extrusion may represent a marker of earlier compartmental narrowing, whereas effusion and osteophytosis appear more prominent once the joint shows a more reactive structural phenotype. This interpretation is hypothetical and would require longitudinal confirmation. Recent ultrasound data support this multiparametric interpretation, showing that osteophytes, synovitis and meniscal extrusion frequently coexist and should be considered interconnected features rather than isolated abnormalities [32]. Taken together, these findings reinforce the value of ultrasound as a complementary tool for characterizing both structural and inflammatory aspects of knee osteoarthritis. In our cohort, meniscal extrusion was most closely associated with earlier medial compartment narrowing, whereas joint effusion showed the strongest statistical association among ultrasound markers in the right knee. This supports the use of a combined ultrasound profile to better characterize the mechano-inflammatory phenotype of knee osteoarthritis [6].

Several limitations of this study should be acknowledged. First, the sample was small and was recruited consecutively at a single tertiary rheumatology center, which limits both the statistical power of subgroup analyses and the generalizability of the findings to unselected populations with knee osteoarthritis. Consequently, several associations reached statistical significance in one knee but not in the other; since the two knees were not formally compared, these differences may reflect sampling variability and limited statistical power. Sex-based comparisons in particular were secondary analyses, and the composition of the cohort (consistent with female predominance) did not allow meaningful comparison between sexes. Second, the cross-sectional design precludes any inference regarding temporal or causal relationship. The associations between joint space narrowing, osteophyte formation, meniscal extrusion and effusion should be interpreted as components of a structural and mechano-inflammatory imaging profile rather than as a defined sequence of disease progression. Moreover, the ultrasound markers compared in Table 2 were not mutually independent, as they identified substantially overlapping subsets of knees; their ranking by *p*-value therefore reflects this overlap rather than distinct structural contributions. Third, no reference standard such as magnetic resonance imaging or arthroscopy was used. The comparison between the two modalities therefore reflects differences in detection rate, with limits regarding its clinical interpretation. Fourth, although ultrasound assessments were performed by two experienced musculoskeletal ultrasonographers using a standardized EULAR-based protocol, with final data recorded by consensus, formal intra- and interobserver reliability analyses were not performed. Similarly, radiographic measurements were performed by a single trained investigator, limiting the assessment of interobserver reproducibility, although blinding to clinical and ultrasonographic data reduced potential interpretation bias. Finally, although inclusion and exclusion criteria were applied to minimize major diagnostic confounders, some patient-related factors, including body mass index, were not included in the comparative imaging analysis. Lower-limb alignment was indirectly reflected by standardized weight-bearing radiographs, but was not quantitatively assessed as a separate variable. Ultrasound examinations were performed in the supine position, whereas radiographs were obtained under weight-bearing conditions; since meniscal extrusion increases with loading, its extent may have been underestimated relative to the radiographic assessment. As no standardized clinical or functional scores were collected, the relationship between imaging findings and symptom severity could not be evaluated.

These limitations suggest several directions for future research. Longitudinal follow-up of the same cohort could determine whether US-detected osteophytes in radiographically negative knees precede the development of radiographic changes and whether meniscal extrusion is associated with later compartment narrowing. Comparison with magnetic resonance imaging as an independent reference standard could help determine whether discordant US findings represent early structural changes or false-positive findings. Larger multicenter cohorts with a formal sample size calculation, a more balanced sex distribution, and a quantitative assessment of lower-limb alignment and body mass index would enable multivariable modeling of the relative contribution of individual US features beyond the bivariate analyses performed in the present study. Finally, systematic assessment of power Doppler activity and synovial hypertrophy, together with validated instruments such as the WOMAC index or VAS, would allow the structural and inflammatory US findings to be examined in relation to symptom burden and functional status.

## 5. Conclusions

Musculoskeletal ultrasonography identified structural and effusion-related abnormalities that were not fully captured by standard weight-bearing radiography in the bilateral assessment of knee OA. Ultrasound detected marginal osteophytes in 86.7% of left knees and 83.3% of right knees, compared with 56.7% and 63.3% by radiography (*p* = 0.003 and *p* = 0.014, respectively). Osteophytes were identified exclusively by ultrasound in 15 knees, and in none by radiography alone, with limited agreement between the two modalities (κ = 0.34 for the left knee; κ = 0.51 for the right knee). Ultrasonographic cartilage thickness was not significantly correlated with radiographic medial JSW (*ρ* = −0.266, *p* = 0.156 for the left knee; *ρ* = 0.092, *p* = 0.628 for the right knee). Medial meniscal extrusion was associated with a 1.94-mm lower median radiographic medial JSW in the right knee (3.79 vs. 5.73 mm; *p* = 0.024), while joint effusion was observed exclusively in knees with ultrasound-detected osteophytes (84.6% and 76.0%; *p* = 0.003 for both knees). Osteophyte severity was significantly correlated between the left and right knees in both the medial (*ρ* = 0.654) and lateral (*ρ* = 0.628) compartments (both *p* < 0.001). Overall, these findings support the complementary role of musculoskeletal ultrasonography in the multiparametric assessment of knee osteoarthritis, providing additional information on osteophytes, meniscal extrusion, and joint effusion that may be underestimated or not detectable by radiography.

## Figures and Tables

**Table 1:** Comparison of radiographic medial JSW between sexes.

Joint/Compartment	Sex	*n*	Median JSW (mm)	*p*-Value (Mann–Whitney U)
Left Knee—(Medial)	Female	24	4.59	0.436
	Male	6	5.12	
Right Knee—(Medial)	Female	24	4.00	0.640
	Male	6	4.25	

Note: JSW = Joint Space Width. *n* = 30 patients. The threshold for statistical significance was set at *p* < 0.05.

**Table 2:** Association between ultrasonographic markers and radiographic medial JSW narrowing.

Knee	Marker (Ultrasound)	*n* (Absent/Present)	Median JSW—Absent, mm (IQR)	Median JSW—Present, mm (IQR)	*p*-Value (Mann–Whitney U)
Left	Medial Meniscal Extrusion	7/23	5.80 (4.20–6.00)	4.58 (3.80–5.40)	0.073
	Joint Effusion	8/22	5.15 (4.38–5.88)	4.56 (3.68–5.42)	0.111
	Medial Osteophyte	4/26	4.45 (4.22–5.62)	4.65 (3.80–5.55)	0.879
Right	Joint Effusion	11/19	5.00 (4.00–6.00)	3.40 (2.90–4.30)	0.001 **
	Medial Osteophyte	5/25	5.73 (4.75–5.90)	3.79 (3.08–4.65)	0.009 **
	Medial Meniscal Extrusion	5/25	5.73 (4.47–5.90)	3.79 (3.08–4.65)	0.024 *

Note: Data are presented as median [interquartile range]. JSW = Joint Space Width. *n* = 30 patients. Statistical significance level: * *p* < 0.05; ** *p* < 0.01. Markers ranked by ascending *p*-value within each knee.

## Data Availability

The data presented in this study are available on request from the corresponding author. The data are not publicly available due to privacy and ethical restrictions concerning patient confidentiality.

## Abbreviations

The following abbreviations are used in this manuscript:

ACR	American College of Rheumatology
AP	Anteroposterior
CI	Confidence interval
EULAR	European Alliance of Associations for Rheumatology
IQR	Interquartile range
JSN	Joint space narrowing
JSW	Joint space width
OA	Osteoarthritis
PACS	Picture Archiving and Communication System
Rx	Radiography
US	Ultrasonography
VAS	Visual Analog Scale
WOMAC	Western Ontario and McMaster Universities Osteoarthritis Index
